# Dental status, oral prosthesis and chewing ability in an adult and elderly population in southern Brazil

**DOI:** 10.6061/clinics/2017(11)06

**Published:** 2017-11

**Authors:** Alexandre Baumgarten, Jeanne Gabriele Schmidt, Rafaela Soares Rech, Juliana Balbinot Hilgert, Bárbara Niegia Garcia de Goulart

**Affiliations:** I Graduate Program in Epidemiology, Universidade Federal do Rio Grande do Sul, Porto Alegre, Brazil; II Graduate Program in Dentistry, Universidade Federal do Rio Grande do Sul, Porto Alegre, Brazil

**Keywords:** Adult, Aged, Chewing Ability, Oral Health, Dental Prosthesis

## Abstract

**OBJECTIVE:**

The objective of this study was to explore the factors associated with inadequate chewing in an adult and elderly population of a city in the southern region of Brazil.

**METHODS:**

This was a cross-sectional study based on a population home-based inquiry (DCH-POP) in southern Brazil. Individuals were interviewed by trained interviewers to create a standardized procedure. In a pilot study, the Questionnaire of Human Communication Disorders (DCH-POP) was created and validated to identify self-reported speech and language, swallowing and hearing disorders. The outcome was dichotomized into either having adequate chewing or not, as assessed by a series of questions about chewing ability. Analyses of absolute and relative frequencies were measured according to the studied variables. A Poisson regression was applied at a significance level of 5%.

**RESULTS:**

A total of 1,246 people were interviewed. Inadequate chewing was found in 52 (5.6%) individuals, with a higher prevalence in the elderly (11.8%) than in adults (5.2%). In the final model, the following factors were associated with inadequate chewing: being 61 years of age or older (prevalence ratio or PR=9.03; 95% CI: 1.20-67.91), loss of teeth and use of unadjusted prosthesis (PR=3.50; 95% CI: 1.54-7.95), preference for foods of soft consistency (PR=9.34; 95% CI:4.66-8.70) and difficulty in nasal breathing (PR=2.82; 95% CI: 1.31-6.06).

**CONCLUSION:**

Age, oral health status through dental prosthesis, preference for foods of soft consistency and difficulty breathing through the nose were factors associated with chewing inability in adults and the elderly.

## INTRODUCTION

Chewing involves neuromuscular and digestive activities. It is considered the most important function of the stomatognathic system 1and indicates the ability to crush, grind and mix food with saliva, as well as the ability to form the bolus 2. Thus, the act of chewing creates a relation of interdependence with nutritional conditions, since impaired chewing can decrease nutritional quality 3,4.

Tooth losses influence chewing function and efficiency 5,6. It has been established that tooth loss is associated with the election of food consistency, difficulty in food deterioration, and poorer chewing ability 7. Although dental prostheses are an artificial substitute for the teeth and may perform a similar function, the use of dental prostheses and/or unadjusted prostheses does not perform a satisfactorily masticatory function, which leads to changing eating habits 3,8.

In some cases, chewing occurs in conjunction with oral breathing, whether or not it is associated with nasal obstruction. In these cases, the activity of the masticatory muscles and the time spent to perform the function are reduced. Breathing through the mouth may reduce the degree and duration of the vertical occlusal force on the posterior teeth and may induce vertical problems in malocclusion 9,10. Thus, prolonged chewing is required to form a bolus and initiate swallowing. Oral breathing restricts chewing in daily life, and it is not uncommon that chewing competes with breathing and may be associated with chewing inefficiency, as well as dentofacial alterations, at any stage of life 11,12.

Chewing, therefore, plays a critical role in the daily lives of all individuals. Previous studies examining this phenomenon usually use clinical or institutionalized populations 8,10,11,13. It is critical to understand the distribution of chewing ability as well as the factors associated with this condition through a population-based household survey to understand the dysfunctions in the adult and elderly population, such as difficulties in eating consistent foods and difficulties in forming the food bolus for swallowing 14,15. It is also critical to have subsidies to propose actions directed towards health promotion and the greatest effectiveness in the scope of prevention, diagnosis and rehabilitation. Thus, the objective of this study was to explore the self-reported factors associated with inadequate chewing in an adult and elderly population of a city in southern Brazil.

## METHODS

### Type of study and sample qualification

A cross-sectional population-based study was conducted in the city of Porto Alegre, Brazil, between 2010 and 2014. This study is part of a household survey of the self-reported Human Communication Disorders Population Study (HCD-POP) 16.

Probabilistic sampling was performed, stratified by multiple stages and determined from an analysis of age distribution and education. For the sample size of 1,500 individuals, a significance level of 95% was used to establish the confidence intervals (z=1.96), with a sampling error of 10% and a proportion of 20% (*p*=0.20) to be estimated in population subgroups. The absolute number of people with human communication disorders was estimated by expanding the sample data to the total Brazilian population in the same age group and geographic area 16. Differences between categories were assessed by the overlap of their confidence intervals.

The criterion of eligibility was to reside at an address in the selected neighbourhood. After selecting the residences, buildings with multiple units had their individuals listed from the lowest floor to the highest floor. All residents in the selected houses were considered eligible for the study, with exclusionary factors of being institutionalized during the collection of data, the occurrence of four or five home visits at alternate times with no answer, as well as unsuccessful telephone contact. One respondent per household was chosen according to his or her willingness to participate in the survey.

For the data collection, interviewers were selected and given uniform training and face-to-face refreshment courses every three weeks to help them recall the methodology of all data collection steps, with the objective of monitoring data collection and improving respondents’ likelihood of participating in the study.

### Outcomes and variables

The outcomes included issues related to chewing and its possible difficulties, dichotomized into either having adequate chewing (AC) or not (inadequate chewing or IC). The chewing was considered adequate upon a negative response to the following items: chewing difficulty, noisy chewing, open mouth chewing, pain during the chewing, difficulty swallowing and cracking during chewing.

A questionnaire was administered consisting of sociodemographic variables for the following information: I) sex (male/female); II) age (in years), categorized into 18-30 years, 31-60 years or 61 years or older; and III) education (in full years), categorized in 0-9 years, 10-12 years and 12 years or more. Additionally, independent variables, which had response options of “yes”, “no”, or “no response”, included information on the following: I) tooth loss; II) use of dental prosthesis (has all teeth, lost teeth and does not use prosthesis, lost teeth and uses fitted prosthesis, or lost teeth and uses unadjusted prosthesis); III) keep their mouth open most of the time; IV) previous speech therapy treatment; V) preference for a specific type of food consistency; and VI) difficulty with nasal breathing.

### Data analysis

Data were analysed using *SPSS v.21* software (Chicago: SPSS Inc). Absolute and relative frequency analyses were calculated, in addition to chi-square tests, independent samples t-tests and Fisher’s exact tests. A *p*<0.05 significance level was used to evaluate differences in the studied variables. A Poisson regression with robust variance was performed to obtain the prevalence ratios (PR) with their respective 95% confidence intervals (95% CI). Three models were created to test the associations between the outcome and the self-reported associated factors.

### Ethical research criteria

This study was approved by the Research Ethics Committee of the Federal University of São Paulo under number 0150/2010. The researchers followed the guidelines set forth in Resolution 496 by the National Health Council.

## RESULTS

Out of the 1,500 subjects predicted in the HCD-POP study, 1,246 individuals were interviewed (losses and refusals: 16.9%), 321 of whom were excluded from this analysis because they were under 18 years old. The other 925 participants corresponded to the population of this study. Women constituted the majority of the sample (58.1%).The mean age was 48.9 (SD ±19.6) years, while the number of years of education was 12.9 (SD ±3.4) years. Inadequate chewing was found in 52 (5.6%) individuals in the sample and was more prevalent in the elderly (11.8%) than in adults (5.2%).

**Figure 1:**
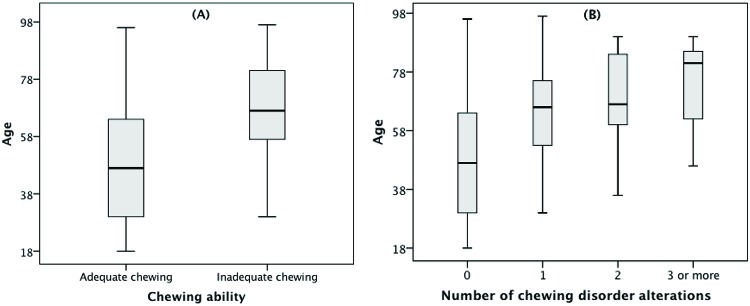
Assessment of age by (A) chewing ability and (B) number of chewing disorder alterations. Porto Alegre, Brazil, 2012.

The proportion of AC in the sample and its associations with socioeconomic, orofacial and anatomical characteristics are presented on Table 1. No statistically significant associations (*p*>0.05) were found between AC and sex or education.IC was more frequently found in older individuals (mean age of 67.2 (SD ±16.5) years) than those who had AC, which was found in individuals with a mean age of 47.8 (SD ±19.3) years (*p*<0.001). In contrast, IC was self-reported more often in individuals who had lost their teeth (65.4%) and who wore dental prostheses (61.5%) (*p*<0.001). As the age of the individuals in the sample increases, an increase in inadequate chewing and number of disorder alterations is also observed (Figure 1).

Table 2 presents a Poisson regression with crude and adjusted prevalence ratios and confidence intervals. After adjusting the final model, some independent variables lost their statistical significance, remaining associated with the outcome only: being 61 years of age or older (PR=9.03; 95% CI: 1.20-67.91), loss of teeth and unadjusted prosthesis (PR=3.50; 95% CI: 1.54-7.95), preference for foods of soft consistency (PR=9.34; 95% CI:4.66-18.70) and difficulty with nasal breathing (PR=2.82; 95% CI: 1.31-6.06). The adjusted model was verified by the χ^2^ Pearson test (*p*=0.864) and omnibus test (*p*<0.001). Subgroup analysis was performed, but no effect modification was identified.

## DISCUSSION

In this study, it was possible to evaluate self-reported chewing alterations of a sample from Porto Alegre, southern Brazil. It was observed that inadequate chewing was found in only 5.6% of the interviewed population. To our knowledge, this is the first study to assess adults and elderly individuals based on data from a population-based survey and a probabilistic sample stratified by multiple stages. It was found that adult and older individuals who wear unadjusted prostheses prefer foods with a softer consistency, have difficulty breathing through the nose and have a higher prevalence of experiencing inadequate chewing.

Few studies have investigated the prevalence of chewing alterations in adult populations. Among individuals aged between 20 and 59 years in Florianópolis (Brazil), the chewing difficulty was 13% for men and 18% for women 17. In a study of elderly individuals aged 65 to 74 years old in 250 cities in all Brazilian states, the prevalence of unsatisfactory chewing ranged from 47.5% to 51.8% 3. In contrast to the findings of this study, the prevalence ranged from 5.2% in adults to 11.8% in the elderly. Some of these differences can be explained by the fact that each study used different methodologies, either in terms of sampling or in the detection of chewing alteration, from self reports to evaluation by an examiner with different degrees of training, which affect the ability to compare differences in prevalence.

In the process of ageing, the stomatognathic system undergoes several physiological changes.These changes may be both neurological and anatomical and may result in decreased neuromuscular activity, reflexes, sensitivity, saliva production, sense of taste, strength and tongue movements. It is also known that with advanced age, masticatory work is less efficient, and the strength employed is lower 18, which may hamper the physiological act of chewing. However, the oral health of the elderly also has a great influence, being strongly related to the presence of cavities, periodontitis, xerostomia, tooth loss and/or unadjusted prostheses 19-21. All these changes, whether associated or not, contribute to inadequate chewing 18,21. The findings of this study indicate that the effect of age had a major influence on the masticatory inadequacy of individuals older than 60 years.

Among the main results presented, the use of total dental prosthesis negatively interferes with chewing and food preference for softer consistency. The total number of teeth is directly related to chewing ability, thus influencing food choices 22,23. The more teeth, the greater the possibility of choosing foods that are not soft or that are difficult to chew 24. Individuals with masticatory difficulties tend to consume less fibre, thus becoming more vulnerable to nutritional deficiencies and gastrointestinal diseases 25. In contrast, tooth loss and masticatory difficulty favour the selection of more easily crushed foods, which generally have fewer essential nutrients, including protein, fibre, vitamin D, niacin, pantothenic acid, vitamins B1 and B6 24,26. In addition, eating habits, especially those related to the consistency of the food ingested, have not been evaluated in studies of chewing ability 3,7,17.

It is well known that there is interference between breathing and chewing, especially when both functions compete with each other 27,28, and this interference becomes even more relevant in colder regions, such as southern Brazil, where people have more respiratory complaints. Studies also show that oral breathing interferes negatively with food leftovers in the oral cavity, lip positioning and noise while chewing 18, although there is no proven association with nutritional status 29. Faced with greater scientific evidence on these changes, the planning of health activities in the early stages of pathological development prior to main clinical manifestations is critical for greater control of these alterations, which strongly impact public health.

This is an original population-based study with data that represent the southern Brazilian population, which has similar characteristics and habits in terms of eating and cultural habits. In addition to the consistency of the findings with those in the literature, the prevalence and associations presented in this study are relevant for the planning of health policies aimed at promoting integral healthcare for adults and the elderly. This study has some limitations. Despite its random sample of individuals at multiple stages of life, women were the main respondents and constituted the majority of the sample, although it is well known that men die more frequently in all age groups than women. However, this factor was corrected in the multivariate analysis. Additionally, a variation in the outcome measure could be expected from the self-report survey. However, the literature is already consistent in stating that for chronic diseases, self-reports are usually reliable 30-32.

Age, oral health status through dental prosthesis, preference for foods of soft consistency and difficulty breathing through the nose are factors associated with masticatory inadequacy in adults and the elderly. Thus, it is extremely important for healthcare teams to carefully investigate and evaluate these factors.

## AUTHOR CONTRIBUTIONS

Baumgarten A, Schmidt JG and Rech RS were responsible for the data analysis, interpretation of results, drafting of the manuscript and critical review of the manuscript. Hilgert JB was responsible for the coordination and critical review of the manuscript. de Goulart BN was responsible for the conception and design of the study, coordination, data collection, data analysis, interpretation of results and critical review of the manuscript.

## Figures and Tables

**Table 1:** Association between adequate chewing and the associated factors. Porto Alegre, Brazil, 2012.

Variables	Inadequate chewing	Adequate chewing	*p*-value
Sex			
Male	17 (32.7%)	370 (42.4%)	0.107
Female	35 (67.3%)	502 (57.6%)	
Age			
In years	67.2 (± 16.5)	47.8 (± 19.3)	<0.001*
Education (years)			
0-9	7 (15.9%)	90 (12.5%)	0.778
10-12	12 (27.3%)	193 (26.7%)	
<12	25 (56.8%)	439 (60.8%)	
Tooth loss			
No	18 (34.6%)	644 (73.9%)	<0.001
Yes	34 (65.4%)	228 (26.1%)	
Use of dental prosthesis			
No	20 (38.5%)	682 (78.4%)	<0.001
Yes	32 (61.5%)	188 (21.6%)	
Keep their mouth open most of the time			
No	45 (86.5%)	836 (96.3%)	<0.001
Yes	7 (13.5%)	32 (3.7%)	
Pre-treatment of speech-language pathology			
No	51 (98.1%)	871 (99.8%)	0.160**
Yes	1 (1.9%)	2 (0.2%)	

Chi-square test (no symbol).

* T-Test for independent samples.

** Fisher's exact test.

**Table 2:** Poisson regression with robust variance of adequate chewing in adults and the elderly. PR=prevalence ratio. Porto Alegre, Brazil, 2012.

Variables	n	Crude PR (95% CI)	Model 1^a^ PR (95% CI)	Model 2^b^ PR (95% CI)	Model 3^c^ PR (95% CI)	*p*-value^d^
Age (years)						
18-30	185 (24.6)	1	1	1	1	
31-60	334 (44.5)	7.70 (1.02-58.14)	6.05 (0.79-46.45)	4.19 (0.54-32.34)	4.22 (0.54-32.68)	0.168
61 or more	232 (30.9)	27.08 (3.74-195.84)	22.56 (3.16-161.12)	9.41 (1.25-70.55)	9.03 (1.20-67.91)	0.032
Sex						
Male	320 (42.6)	1	1	1	1	
Female	431 (57.4)	1.48 (0.84-2.61)	1.18 (0.64 - 2.19)	1.01 (0.58-1.77)	1.15 (0.65-2.03)	0.627
Education (years)						
0-9	93 (12.4)	1	1	1	1	
10-12	200 (26.6)	0.81 (0.33-1.99)	0.92 (0.38-2.23)	0.87 (0.38-1.98)	0.62 (0.24-1.62)	0.329
<12	458 (61.0)	0.75 (0.33-1.68)	0.97 (0.44-2.11)	1.06 (0.51-2.21)	1.12 (0.59-2.24)	0.753
Oral Health						
Has all teeth	553 (73.6)	1		1	1	
Lost teeth and does not use prosthesis	46 (6.1)	2.58 (0.90-7.37)		1.49 (0.52-4.20)	1.56 (0.56-4.35)	0.397
Lost teeth and uses adjusted prosthesis	123 (16.2)	2.81 (1.39-5.71)		1.71 (0.83-3.52)	1.99 (0.99-4.00)	0.054
Lost teeth and uses unadjusted prosthesis	30 (4.0)	16.71 (9.27-30.13)		3.71 (1.68-8.18)	3.50 (1.54-7.95)	0.003
Preference for foods of soft consistency						
No	738 (98.1)	1		1	1	
Yes	14 (1.9)	26.68 (19.18-37.10)		10.15 (5.37-19.16)	9.34 (4.66-18.70)	<0.001
Difficulty breathing						
No	670 (89.2)	1			1	
Yes	81 (10.8)	3.51 (1.70-7.28)			2.82 (1.31-6.06)	0.008

a Adjusted for age, sex and education.

b Adjusted for model 1, oral health and preference for soft consistency food.

c Adjusted for model 2 and difficulty breathing through the nose.

d *p*-value model 3.
